# Public-engagement strategies of the South Asian COVID-19 Task Force: The role of racialized healthcare workers in COVID-19 mitigation in Ontario

**DOI:** 10.1371/journal.pgph.0003729

**Published:** 2024-10-02

**Authors:** Pushpita Samina, Chandrima Chakraborty, Rajdeep Grewal, Tajinder Kaura

**Affiliations:** 1 Centre for Health Economics and Policy Analysis (CHEPA), McMaster University, Hamilton, Ontario, Canada; 2 Department of English and Cultural Studies and Population Health Research Institute, McMaster University, Hamilton, Ontario, Canada; 3 Faculty of Health Sciences, Department of Family Medicine, Division of Emergency Medicine, DeGroote School of Medicine, McMaster University, Hamilton, Ontario, Canada; University of the Southern Caribbean, TRINIDAD AND TOBAGO

## Abstract

The COVID-19 pandemic began in late 2019 and its uneven impact across different communities globally was quickly evident. In Canada, South Asian communities were disproportionately affected. In response, the South Asian COVID-19 Task Force (SACTF) emerged, seeking to address the unique challenges faced by the South Asian community. The embedded single case study design was employed to explore the role of SACTF in COVID-19 mitigation in Ontario. Informed by critical race theory and a public engagement conceptual framework published by the Canadian Health Services Research Foundation (2010), we analyzed how contexts guided the goals, processes, and outcomes of SACTF activities. We conducted one-on-one semi-structured interviews and focus group discussions with SACTF’s Board of Directors and analyzed SACTF-produced knowledge dissemination materials and media coverage of SACTF spanning March 2020 to February 2022. SACTF’s success in educating and advocating for South Asians offers important insights into the gaps in public health communication and the inequities in healthcare delivery. It emphasizes the importance of tailoring emergency responses to community-specific needs and the role of racialized healthcare workers in facilitating trust-building within minority communities. By incorporating insights of racialized healthcare workers in health system decision-making, both public engagement and community health outcomes can be improved. This study contributes to a nuanced understanding of community-centric pandemic responses and demonstrates the need for diverse representation in decision-making processes for long-term health system resilience. Both healthcare knowledge and lived experiences made SACTF alert to how pandemics unfold differently and have differential effects on racialized populations. SACTF’s responses offer practical recommendations for future pandemic preparedness and emergency responses, emphasizing the role of advocacy groups in addressing public health gaps and serving as crucial allies for communities and governments.

## Introduction

The COVID-19 pandemic has revealed the intricate interplay between public health crises and policy decision-making, posing unprecedented challenges for governments worldwide [1]. As governments grappled with the urgent need to formulate effective policies amidst uncertainty, the importance of public engagement in shaping these policies came to the forefront [2]. Historically, public engagement in public health policymaking has gained prominence since the 1978 Alma Ata Declaration, which emphasized the social determinants of health and the need for community involvement [3]. Subsequent international efforts, such as the International Health Regulations of 2005, underscored the importance of working with communities to effectively respond to disease outbreaks on a global scale [2]. Despite these efforts, the full potential of public engagement in pandemic policymaking has not always been realized.

Simultaneously, the COVID-19 pandemic showed how longstanding inequities and disparities within global health systems took a much larger toll on vulnerable communities worldwide. Racial minorities, religious minorities, older adults, those of low socio-economic status, or those residing in smaller communities often bore a much higher burden of infections and deaths from COVID-19 [4]. Like many other countries, Canada also struggled in handling the pandemic, and the pandemic took thousands of lives in Canada. In addition, available data demonstrates that the pandemic disproportionately impacted racialized groups in Canada [5]. While public health approaches to tackle the high COVID-19 incidence were in place, they were not comprehensive enough to address the specific needs of racialized minorities [6]. Research has demonstrated that those who experience racism have reduced trust in healthcare systems and healthcare professionals, and compromised communication and relationships with healthcare providers reduce their adherence to medical regimens [7]. A similar trend was seen among racialized communities in Canada, including South Asian populations, which demonstrated distrust in public health communications and lack of compliance to public health guidelines at the beginning of the pandemic. South Asians immigrants, predominantly coming from India, Pakistan, Bangladesh, and Sri Lanka, and their descendants [8] are the largest non-white ethnic group in Canada, and immigration from India to Canada is rapidly increasing [9].

The province of Ontario, which saw a high incidence of COVID-19, is home to the largest South Asian Canadian communities in Canada. In Ontario, immigrants represent about 25% of the population but reported 43.5% of COVID-19 cases, of which most were racialized minorities [10]. Studies indicate that South Asian and Black communities in the Greater Toronto Area (GTA) experienced the largest rate of positive COVID-19 cases [11]. In January 2021, South Asians had the highest rate of COVID-19 infection in Toronto (27%) despite making up only 13% of the population [12]. Other South Asian-dominated regions, such as Calgary (Alberta) and Surrey (British Columbia), experienced similarly high infection rates. The vulnerability of South Asians to COVID-19 can be attributed to factors such as precarious employment, poor, overcrowded or multigenerational housing, and pre-existing medical conditions [13,14]. Misinformation, lack of accessible public health information, and difficulty navigating public health protocols further increased their vulnerability during the pandemic, especially with public health guidelines changing quickly as the pandemic evolved [15].

In this context, various South Asian advocacy groups and non-profit organizations mobilized to help their community to curb the spread of infection. Advocacy groups, researchers, and community representatives played a critical role in directing attention to the vulnerabilities and specific needs of South Asian populations. They helped the community overcome some of the structural barriers and follow public health guidelines [16,17]. Their collective efforts resulted in the South Asian community to become the highest-vaccinated (96%) ethnic minority group that received at least one dose of the COVID-19 vaccine between June 2021 and February 2022 [18].

**Fig 1:**
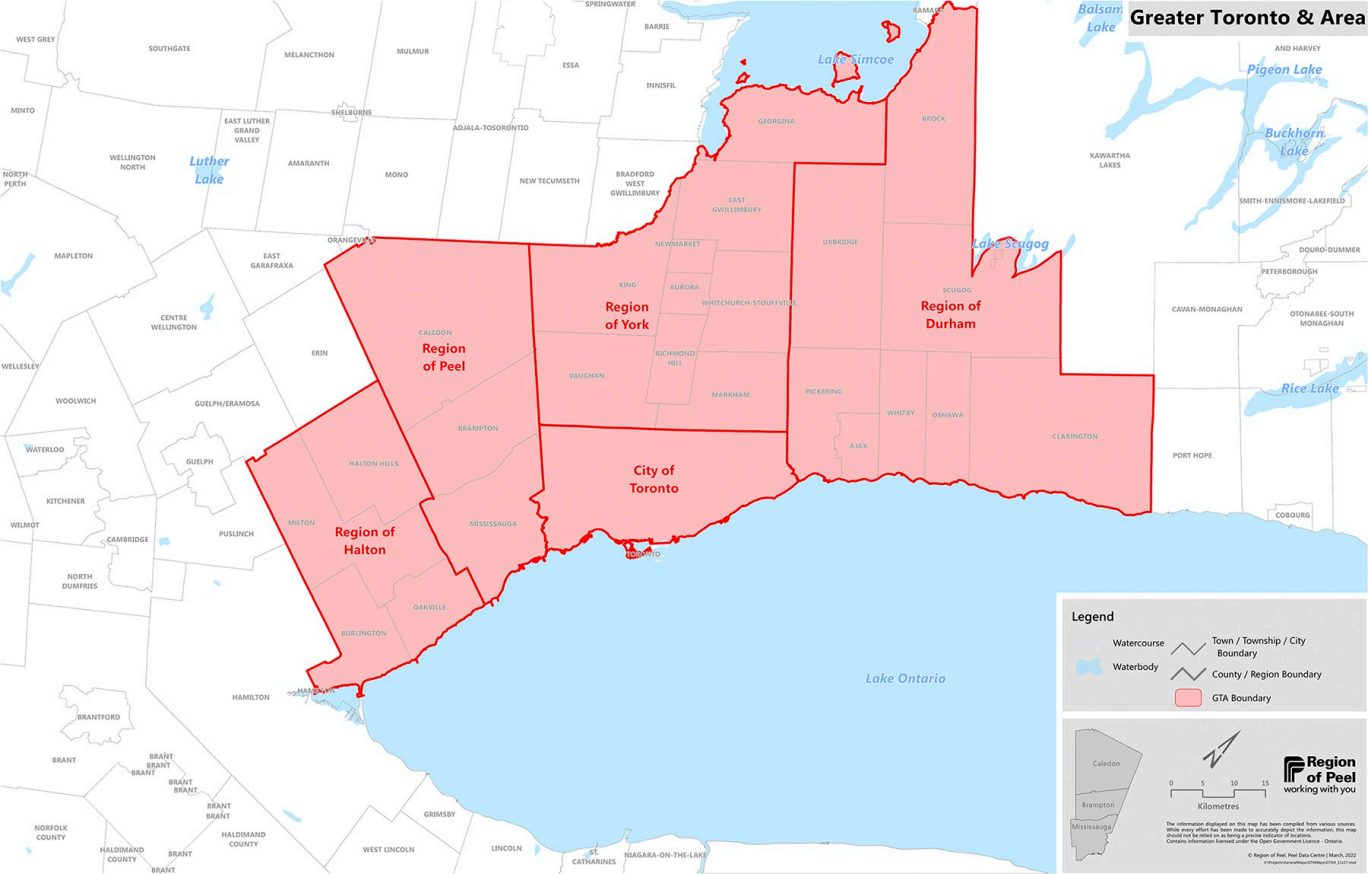
Map of Greater Toronto Area.

South Asian advocacy groups established multiple foci, including educating South Asian communities about COVID-19 and vaccination, vaccinating South Asians against COVID-19, and informing the COVID-19 public health response towards equity for South Asians. However, these efforts and the impact of this multipronged work have not been formally studied [19]. The South Asian COVID-19 Task Force (SACTF) was an advocacy group formed by a group of physicians in November 2020, in direct response to the disproportionate numbers of South Asians affected adversely by the COVID-19 pandemic. Their goal was "to educate and advocate for the community and reduce the spread of this virus" [20]. Our study explores the public engagement strategies of SACTF to understand the public health gaps that motivated the formation of SACTF, how SACTF mobilized to meet the distinct needs of South Asians during COVID-19, and the outcomes they produced in allaying the disproportionate burden of COVID-19 on South Asians. Specifically, we explore how SACTF’s strategies and approaches bridged gaps in equity-focused public engagement among South Asians in the Regional Municipality of Peel, in the Greater Toronto Area (GTA; see Fig 1), which was declared a nationwide pandemic hot spot, with the City of Brampton as the epicenter [21]. Peel is Ontario’s second-largest municipality, with a population of almost 1.5 million [22]. More than half of the residents in the Peel region identify as South Asian [9]. 48% of recent immigrants to Peel live in Brampton [23].

This study reveals how the insights and experiences of racialized healthcare workers who formed SACTF contributed to the development and implementation of effective public health strategies targeted toward equity-seeking groups. Drawing from the lessons learned from SACTF’s approach, we provide recommendations that can improve healthcare access and delivery to minority groups. The overarching goal is to address structural global health inequities and improve health outcomes for marginalized communities.

## Methods

### Theory/Framework

There is a long history of unethical medical and scientific experimentation on racialized peoples in the West that fosters distrust of the healthcare system within minority communities [24,25]. In addition, South Asian and East Asian immigrants have been historically targeted in Canadian health discourse [26,27] and public health campaigns [28,29]. In the early twentieth century, Canadian and US public health authorities portrayed Indian and Chinese immigrants as carriers of plague and other infectious disease that threatened the health of white populations, and this then became the rationale for implementing exclusionary immigration, housing, and public health policies [27,28]. This intertwining of race and health in Canadian history needs to be explicitly considered in order to address issues of mistrust of healthcare messaging, misinformation, and vaccine hesitancy during the COVID-19 pandemic. Therefore, we adopted critical race theory (CRT) to understand better the context that prompted SACTF formation and public engagement. CRT integrates theory, experiential knowledge, and critical consciousness to illuminate and combat the root causes of structural racism. It recognizes that race is socially constructed, not biologically natural. Race is socially significant because racism is experienced in the everyday; it is structural, embedded within systems and institutions and woven into public policy. In addition, CRT acknowledges that the lived experiences of racialized people are critical to scholarship Therefore, through our interviews with South Asian healthcare professionals who formed SACTF, we tried to uncover the ways in which racial inequities in healthcare impacted South Asian communities in Brampton during the COVID 19 pandemic and SACTF’s public engagement efforts to support their community during this challenging time.

Our alertness to historical and contemporary contexts aligns well with the public engagement conceptual framework published by the Canadian Health Services Research Foundation [30]. This framework emphasizes the significance of context in guiding the goals, processes, and outcomes of public engagement. Contextual factors include, but are not limited to, historical and socio-political situation, characteristics of the community, organizational structure, and leadership or decision-making processes. Building on this framework, we analyzed SACTF’s formation and how its public engagement strategies bridged processes and outcomes.

### Study design

We conducted a qualitative, multimethod, single case study of SACTF, drawing from scholarly literature, document reviews, key informant interviews, and focus group discussion [31]. The embedded single case study design was used to address the research questions as it can explore a particular event extensively within its authentic context, facilitating a detailed analysis of the subject matter using multiple sub-units of analysis [31].

### Study participants and setting

The single case study design and conceptual frameworks required keeping the perspectives of marginalized voices at the center of our analysis. Our goal was to gather perspectives from the SACTF Board of Directors, who worked on the frontlines of the pandemic in the GTA in Ontario and Surrey in British Columbia. SACTF Board of Directors reviewed and provided input on research design and conceptualization of the study and offered us access to SACTF produced materials such as advocacy letters and promotional materials. We shared our findings in the form of a report with SACTF Board of Directors to ensure data validity and avoid any factual errors. They did not participate in data analysis and interpretation.

Recruitment for this study began on November 15, 2022 and ended on April 3, 2023. We conducted eleven interviews with SACTF members; four were female, and seven were male. Ten of them were physicians, and one was a lawyer. Most (eight) were in Ontario; the rest were in British Columbia. The physicians worked in different capacities, including family physicians, emergency physicians, and specialists in Ontario and British Columbia. They have been working as physicians for three to twenty years. They were all Canadians of South Asian origin and familiar with the Canadian healthcare system and South Asian cultural norms and practices.

### Development of an interview guide

Before developing the interview guide, a scoping review was conducted. The interview guide comprised semi-structured open-ended questions designed to capture any information SACTF Board members deemed important or wished to add beyond the interview questions. The focus group was facilitated with minimal intervention from the interviewers, allowing participants to openly share their experiences, contribute to the conversation freely, and prompt each other with questions or comments.

### Data collection procedures

Document review: We conducted a literature review of South Asians and public health discourse to understand the historical legacies of health inequities, the illnesses that predominate in South Asian populations, and healthcare system efforts to improve access to healthcare services. Additionally, we reviewed COVID-19 media coverage of Ontario newspapers in English (2020–2022) to understand the contemporary social, cultural, and economic contexts of racial health inequities. These literature reviews helped the PI (Chakraborty) to develop topics and questions for the interviews and the focus group discussion. Further, the research assembled various materials generated by or about SACTF from November 2020 to February 2022. This time frame encompasses the various stages of the pandemic as COVID-19 interventions evolved (e.g., social distancing, masking, mass testing, vaccination, etc.).

Key informant interviews: We conducted one-on-one interviews and a focus group for this study. One-on-one semi-structured interviews (eleven) were conducted with SACTF’s Board of Directors to gain their perspectives and insights into the organization’s formation, motivations, activities, and perceived impact. One focus group was conducted with SACTF’s Board of Directors to validate the collected data and gain a more in-depth understanding. It allowed us to observe the group dynamics and add to the information we had gathered through the one-on one interviews.

All interviews were recorded on the Zoom platform using McMaster’s institutional Zoom license. Soon after the interviews, interview data was migrated to MacDrive, McMaster University’s secure collaborative cloud workspace. Interviews were transcribed using Trint software and then manually checked for errors. Each interview took approximately 45–90 minutes, and the focus group was held for 90 minutes. To ensure interviewee anonymity, no names or personal data were requested in audio recordings, and we assigned interviewees a numerical ID code for audio recordings, transcripts, and study outputs. The interviewers, who were PhD students at McMaster University, did not have any reporting relationship with the interviewees. One of the interviewers is a clinician familiar with clinical and public health practices related to COVID-19 and was able to verify the clinical information.

### Ethical consideration

The interviews and the focus group were conducted after receiving ethics approval from McMaster University’s Research Ethics Board and written informed consent from the research participants. All procedures performed in studies involving human participants were in accordance with the ethical standards of McMaster Research Ethics Board (Reference number 5958) and with the 1964 Helsinki Declaration and its later amendments or comparable ethical standards.

### Data analysis

The study aimed to identify recurring themes, patterns, and codes within the interview transcripts by employing thematic analysis. The qualitative data analysis process involved several key steps, including data familiarization, coding (NVivo), theme development, and data validation. Two research assistants transcribed the data, and along with the PI, they read and re-read the data to become familiar with its context and content. The research assistants used inductive coding method to code each interview transcript. The preliminary codes summarized the data, helping to identify recurring themes and patterns that aided us in understanding data saturation. The PI was consulted to refine the codes. The varied disciplinary training and lived experiences of the coding team strengthened the analysis as the research assistants interpreted the data differently and contributed to widening the codes and themes that were developed. When the two coders did not agree on specific codes, interpretations were checked against the data to clarify the definition of the codes. An initial codebook was developed to ensure reliability and consistency among the two coders. The iterative thematic analysis approach altered the codebook as new patterns in the data were found. In consultation with PI, codes were refined, combined, and assembled into themes as the analysis progressed. Themes were verified across other research materials and then linked to the research questions. Later, categories and sub-themes were added. The key themes of the analysis include SACTF’s formation, the motivation behind it, its core activities, and their impact.

### Reflexivity

Most of the researchers involved in data collection and analysis are of South Asian origin. While shared cultural background provided valuable insights and facilitated rapport with participants, it may have introduced bias in the analysis and interpretation of findings. Despite our efforts to maintain objectivity and rigor, through the incorporation of insights from multiple disciplines (for example, public health, anthropology, and cultural studies) and double-coded data, researchers’ perspectives and experiences do shape study outcomes.

### Limitations

One limitation of our study is the unavailability of race-specific data from the Peel region. The lack of comprehensive demographic data disaggregated by race has limited our ability to fully assess the impact of SACTF’s initiatives on different racial and ethnic groups in the Peel region. Additionally, our study primarily focuses on the perspectives and experiences of the SACTF Board of Directors. While their insights provide valuable insights into the organization’s activities and perceived impact, and we have corroborated the interview and focus group data with media coverage of SACTF activities and impact, we recognize that the perspectives of the broader public and community members are essential for a comprehensive understanding of SACTF’s role in public health engagement.

## Results

**Fig 2:**
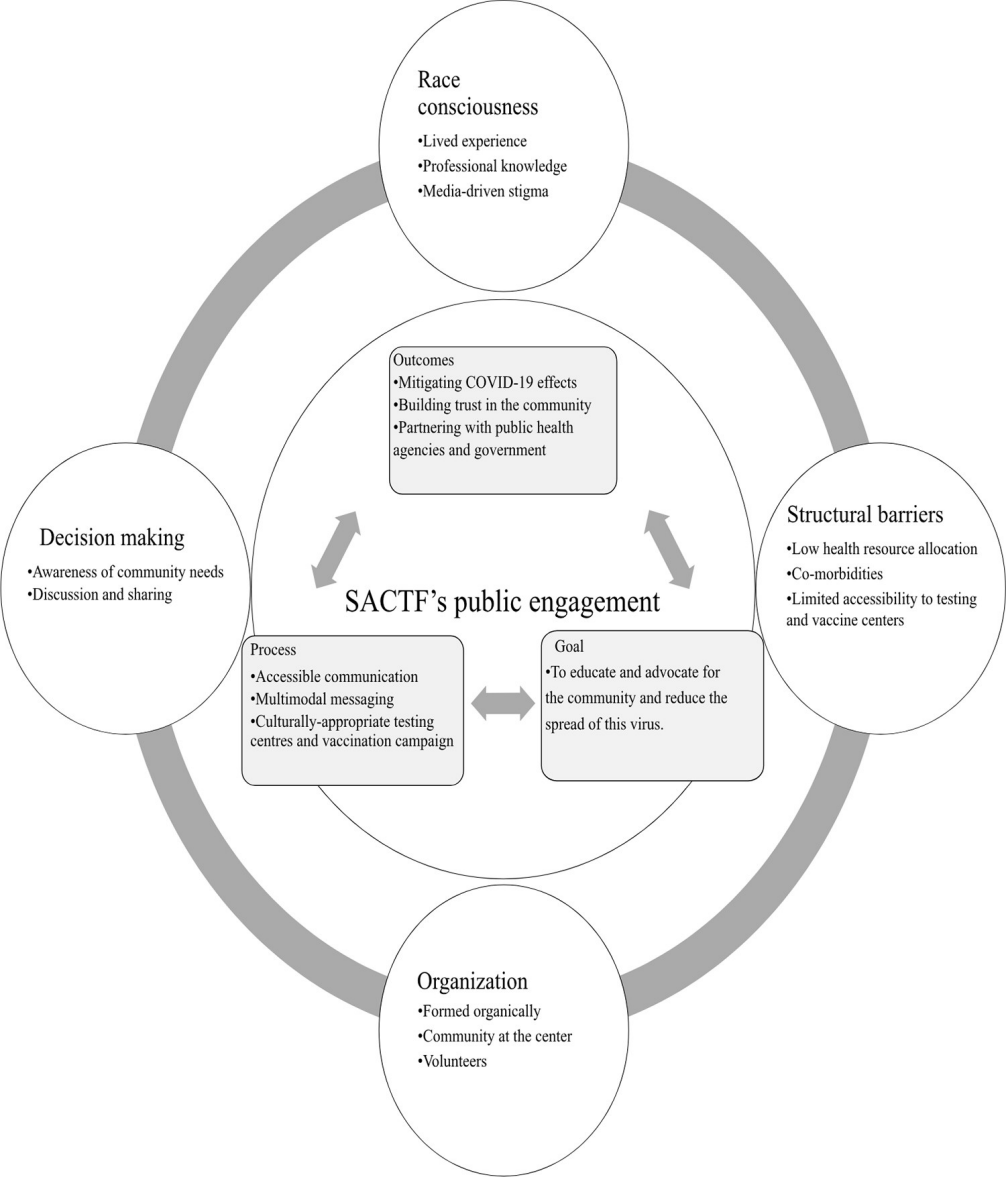
A framework explaining contexts that guided goal, process and outcomes of SACTF’s public engagement.

This study of SACTF’s public engagement during the COVID-19 pandemic identified three primary themes: contexts for SACTF’s public engagement, SACTF organization and goals, and bridging between process and outcome for public engagement. The context for SACTF’s public engagement was further categorized into two sub-themes: racial consciousness and structural barriers. Additionally, bridging between process and outcome for public engagement was divided into sub-themes on process and outcomes. We have structured the themes in accordance with Fig 2.

### Theme 1: Contexts for SACTF’s public engagement

#### Sub-theme 1: Race consciousness

Race consciousness involves individuals recognizing and understanding the implications of their racial identity, including the historical and social factors that shape their societal experiences. Interviews with SACTF members made it clear that the they recognized the systemic discrimination based on race that was producing adverse outcomes during the pandemic for Blacks, South Asians, and Indigenous peoples in Canada. Prompted by their lived and professional experiences, South Asian medical professionals formed this volunteer grassroot organization to tackle the challenges posed by the COVID-19 pandemic in their ethno-religious communities. As South Asian Canadians, SACTF members were aware of the racial discrimination and biases towards their community, and they could quickly identify the media-driven stigma directed towards South Asians anticipating large Diwali (religious festival) gatherings in 2020. Recognizing that "*finger*-*pointing*" in media articles (24110) was eliciting "*discrimination*," "*prejudice*," and "*hatred*" (32512) against South Asian communities, SACTF went on a "*media blitz*" (24110) soon after their formation to counter the blaming of South Asian populations for the increased positivity rates in Brampton and the Peel region more broadly, and to raise awareness about pandemic healthcare guidelines within the South Asian community. As one participant says, it seemed as though it was the South Asian community "*that was failing to follow Public Health guidelines*, *rather than Public Health being able to connect with our community [South Asians] more directly" (28489)*.

Many of the SACTF members had grown up in the region, worked there, and maintained close ties with the community. Both healthcare knowledge and lived experiences made them alert to how pandemics have differential effects on racialized groups, and, as such, they were well positioned to promptly identify and address the vulnerabilities of the South Asian community in the Peel region. For example, many South Asians in Brampton reside in multigenerational homes, which reflects South Asian familial norms where the younger generation is expected to care for and support the elderly in their homes. In addition, when examining the occupational distribution within the workforce, the largest group in Brampton consists of trades and transportation workers, representing 23.38 percent of the workforce. Following closely behind are workers in sales and service, comprising 23.13 percent of the workforce [32]. All these jobs were considered *"essential work"* throughout the pandemic lockdowns, meaning these workers were at a higher risk of COVID-19 infections. The intricate dynamics of multigenerational households, where several family members were also engaged in essential frontline jobs, rendered isolation impractical, and at the time, testing centers were not accessible in terms of location or hours of operation. Those infected with COVID-19 were also resistant to go to the early isolation centers, as the isolation centers did not cater to the unique needs of South Asians, with many serving primarily non-vegetarian food (such as hotdogs) and preventing folks from having contact with their families (61907). One interviewee noted that this "*one size fits all strategy*" was initially "*why Public Health is losing the battle in Brampton*" against COVID-19 (61907).

#### Sub-theme 2: Structural barriers

The pandemic both unveiled and aggravated the many structural inequities that existed in South Asian dominant areas. For example, South Asian-predominant Brampton already had "*per capita […] lower health resources allocated*" to them compared to other areas of the country pre-pandemic (32512). The provincial average for hospital beds is 2.19 per 1000 residents; in Brampton, it is 0.96 [33]. Brampton’s healthcare system could not keep up with *"the fastest-growing immigrant population in Canada*" (27064), and especially with Brampton emerging as a "hotspot" during the pandemic, many more patients needed hospitalization, and many had to be moved to hospitals outside the region. In addition, health conditions prevalent in South Asian communities increased the risk of morbidity and mortality from COVID-19 [12]. As one participant notes, "*We [South Asians] have some of the higher risks of diabetes […]*, *we have the higher risk of high blood pressure*, *we have high risk[s] of obesity*," which creates the "*perfect storm for COVID*" morbidity and mortality (42059). Their knowledge of the pre-existing inequities in healthcare funding in the region of Peel and the underlying health conditions of many in their ethnocultural group allowed SACTF physicians to offer a more nuanced perspective on the higher prevalence and mortality of COVID-19 among South Asian communities.

SACTF also recognized the challenges faced by a diverse community of South Asians in following public health guidelines. SACTF members noted that it was "*tough to book an appointment*" at the testing centers because of the availability of services in only English and French (88246). In addition, the "*confined hours*" (27064) of the testing centers did not complement the work schedule of essential workers, who make up a significant proportion of Brampton’s workforce. Financial constraints and lack of paid time off to get tested also prevented many from utilizing the testing centers (27064). One participant observed, "*They have no choice*. *There was no time off work*. *If you are sick*, *you do not work*, *you do not make money*, *you do not eat*" (61907). The testing centers were also difficult to access for many South Asians (e.g., newcomers, international students, seniors, and precariously employed) who relied on public transportation (32512).

#### Theme 2: SACTF organization and goals

SACTF was formed to alleviate the disproportionate burden of COVID-19 on South Asians. By just "*word-of-mouth*" (42059, 83111, 24110), a group of South Asian healthcare professionals formed this advocacy group. They started their activities with a clear goal of mitigating the impact of COVID-19 and described their work as "[*giving*] *back*" to the community (32512).

Our analysis considered several potentially influential contextual factors that may have impacted SACTF’s COVID-19 response. These included the relationships among the founding Board members, the frontline experience of the Board members, their existing relationships within the community, and the resources (time and funds) available to such grassroots initiatives. The interviews revealed a heightened sense of responsibility, driving individuals with shared goals to come together and support their community during the COVID-19 crisis. Whether familiar with each other through workplace interactions or strangers who had never crossed paths, these individuals, self-identifying as "young South Asians" (42059) and "*physicians committed to educating our community about testing*, *isolation*, *and vaccination*" (88246), banded together to educate their community and advocate for their community during the pandemic.

At the initial stage, there was no formal structure of SACTF. However, as the organization grew and recruited more members, they soon felt the need for structural leadership. A steering committee was established for operational strategy, financial decisions, external communications, and stakeholder engagement. With no financial support to hire dedicated personnel, the steering committee had to meet frequently "*to evaluate [SACTF’s] objectives and goals*" as the COVID-19 pandemic rapidly progressed (88246). The steering committee had informal online meetings daily, and meetings with volunteers were held either weekly or biweekly. They communicated through Zoom and, more frequently, through messaging platforms such as WhatsApp and Slack. They created small internal group chats for steering committee communications and other channels for specific divisions’ work (such as social media and advocacy) on these platforms. Through ongoing discussions, they identified the community’s needs and accordingly decided on social media content, activities, and external stakeholder engagement.

### Theme 3: Bridging between process and outcome for public engagement

#### Sub-theme 1: Process

Alert to the broader socio-economic determinants of health, SACTF recognized the various factors that were exacerbating the adverse effects of COVID-19 among South Asian communities, and they took proactive measures to address these distinct challenges:

a) Accessible communication

In response to the gaps in public health communication, SACTF produced "*user-friendly*" educational videos and infographics to translate public health guidelines (32512). Ensuring they were "*very relatable and culturally sensitive to the South Asian community*" facilitated public outreach (32512). Additionally, videos created in South Asian languages broke down language barriers and were "*more relatable than watching … a [TV] commercial or going to a website on a government site*" (83111). One participant noted that SACTF sought to convey their medical expertise in "*digestible pieces that people would understand*" (83111). SACTF was not changing the messaging" (40945); instead, SACTF was connecting Public Health information to everyday scenarios that were "*applicable to their [South Asian Canadian] lives*" (32512).

b) Multimodal messaging

SACTF utilized ethnic and mainstream media platforms simultaneously to disseminate COVID-19 information. Ethnic media proved to be "*incredibly useful*" (88246) in reaching those in the South Asian community who do not watch mainstream Canadian television. One participant mentioned that SACTF was successful in reaching the South Asian trucking community through Punjabi radio stations as they "*do not listen to the [Western] morning radio shows*,*"* and the older generations of South Asians ("*grandparents*"), who "*watch [their] religious programming*" every Sunday (40945). SACTF also appeared in mainstream anglophone media to reach those South Asians who tuned into these channels and tried to dispel the negative portrayal of South Asians as responsible for increasing infections. Participant 42059 summarizes, "*You go on The National and Global TV and talk about*, *"Hey*, *this is what you said about the community*, *but this is not true*. *This is actually what’s happening; we’re essential workers*, *and we cannot lock down*. *Where do you want us to stay*? *Who is going to run your groceries*? *Who’s in your Amazon deliveries*? *Who’s in a delivery Uber*? *Who is your security guard at your condo board*? *Because we’re all South Asians*!*"*

In addition, SACTF actively utilized various social media platforms to reach different demographics, including the inception of a WhatsApp channel to disseminate information within the community [34]. Recognizing the *"need to have influencers among [their] own community*" (61907) to effectively educate the community about COVID-19 infections and public health protocols to keep themselves and their loved ones safe, they organized town halls and partnered with religious institutions to foster community trust and improve Public Health outcomes (42059).

c) Testing centres

SACTF successfully advocated for opening more accessible testing centres in Brampton and the Peel region, even though Ontario Health considered closing existing clinics due to lack of use. SACTF aided with hiring and setting up new testing sites with "*people that speak [South Asian] language[s]*" and "*look like the people of the community*" (83111) to establish community trust and promote better community engagement. They additionally ensured that all information–including but not limited to signage at the testing centre, the booking website, and pamphlets–was available in as many South Asian languages as possible.

d) Vaccination campaign

To dispel vaccine-related conspiracy theories and engage with vaccine-hesitant South Asians, SACTF held live sessions on social media platforms to address community questions in real time. They adopted a patient-centric approach, avoiding negative stereotypes and listening with patience to those on the fence about vaccination. They set up clinics in familiar and safe community spaces, offered incentives, and addressed language barriers with the aim of dispelling fears and increasing vaccination rates. For example, SACTF helped advocate for a testing site and, eventually, a community-trusted vaccine site at the Embassy Grand Banquet Hall in Brampton because it was a familiar community gathering place for weddings and festivals. The wider community trusted the testing and vaccine sites as they encountered "*the same sort of faces*" (32512) they would have met at a South Asian wedding/festival but with medical backgrounds. Other spaces were also used, such as places of worship and culturally appropriate clinics in collaboration with other community groups. The use of familiar/comfortable community spaces and the shared ethnicity of SACTF doctors and South Asian community members allowed SACTF to establish credibility and build trust within the community.

Through both public education campaigns and the opening of South Asian-run vaccination/testing sites, SACTF was able to reach South Asian communities that initially "*had a lot of distrust towards [the] health system*" (42059) and demonstrated vaccine hesitancy.

The success in convincing the South Asian population to get vaccinated in Brampton and Peel inspired the establishment of the "This Is Our Shot!" campaign, turning it into a nationwide effort. Several SACTF members emphasized how the campaign, reaching beyond South Asians, became a collaborative, national endeavour to encourage COVID-19 vaccination. It involved outreach to other communities such as Black, Indigenous, and Latin American physician groups to collectively spread awareness and build the public’s confidence in getting vaccinated (83111, 24410).

#### Sub-theme 2: Outcomes

SACTF’s approaches and strategies of public engagement addressed many of the gaps in public health communication and the inequities in healthcare delivery. They produced substantial outcomes at various levels, as detailed below:

a) Building trust in the South Asian community

The members who took the lead in creating SACTF were physicians who had witnessed firsthand the escalating rates of South Asians hospitalized and dying of COVID-19 (42059). Their firsthand experience of the disproportionate impact of the pandemic on South Asians and knowledge of the unique circumstances and needs of their community prompted them to directly engage with the community in order to quickly "*establish… a circle of trust within the community*" (42059). They conducted townhalls (virtual and ethnic media) gathering SACTF medical professionals and other trusted experts in the field to answer questions and concerns about COVID-19 posed by community members. SACTF members were alert to the importance of having *"people that looked and spoke like the [South Asian] community that [they] were serving"* (83111) to cultivate trust within the community. One participant explained, "*The fact that we looked like them […] made it very easy for people to trust that what we were telling them to do was the right thing*" (83111). SACTF members agree that their direct outreach to the community to hear their questions and offer real-time answers was critical in garnering community trust.

b) Mitigating the effects of COVID-19

Having culturally appropriate and contextually informed health messaging centring on the South Asian experience was considered critical to COVID-19 mitigation by SACTF. One member mentioned, "*Because I think part of it is not just translating words*, *but also understanding how to communicate in that language in a way that is accessible by that community*. *It is not just*, *you know*, *academic level word to word translation*, *or like a context*" (28489). Through SACTF’s efforts, "[*South Asians]*, *which [were] one of the hardest hit communities in Canada [with COVID-19] ended up at the end of the pandemic being one of the most vaccinated*" (32512). SACTF members also believe and take great comfort in noting that the opening of clinics that increased testing, vaccination, and detection of infections "*saved lives*" (88246). One of the SACTF members noted that nearing the end of the pandemic, with SACTF’s painstaking outreach work, they saw "*almost […] no South Asians coming in with COVID-19*" (42059).

c) Partnering with public health agencies and government

SACTF became advocates for South Asian communities during the pandemic by persistently pushing for resources to be directed to alleviate the disease burden in the Peel region. They lobbied for more testing centres and, eventually, vaccine clinics in the Brampton area. Initially, government agencies hesitated to trust this new grassroots organization that had emerged during the COVID-19 pandemic. However, once it became evident that SACTF was aiding in public health messaging by disseminating critical information in culturally appropriate formats for South Asian communities and, in effect, contributing to public health outreach efforts, they had the full support of public health offices and various levels of government.

The work of SACTF drew the attention of public health authorities, the health ministry, and the media, including several discussions with the Prime Minister’s office to evaluate SACTF initiatives and explore avenues for collaboration with Public Health and other government agencies (27064, 40945). Affirming the need for such partnerships, one interviewee noted, "*We want to sit at the right tables to talk to people*… *To sit at the right tables where decisions are happening and help them*, *help our community better*" (42059).

## Discussion

The term “racialized’ is the commonly accepted term in Canadian public discourse for non-white populations. It is slowly replacing terms like “visible minority” and “ethnic minority” to draw attention to minorities being perceived as being socially different from the racial majority in Canada [35]. We draw upon some of the key tenets of CRT, such as race consciousness, structural discrimination based on race, intersectionality, and the lived experience of racialized peoples, to anchor our discussion of SACTF’s work. Our study reveals that young South Asian healthcare workers who created SACTF identified racial inequities as contributing significantly to the disease burden of South Asians during the COVID-19 pandemic, and their race consciousness prompted them to try to alleviate the adverse outcomes of the pandemic on their community.

SACTF advocated tirelessly for South Asians and was successful in tailoring emergency health responses to serve the specific needs of their community and fostering trust through diverse community engagement initiatives. SACTF’s multipronged efforts in community health advocacy shifted the media representation of South Asian populations as vaccine-hesitant or vaccine-resistant. By highlighting the reasons behind the initial lower use of testing centres in the Peel region, SACTF ensured that much-needed resources were directed to Brampton, resulting in increased testing and later increased vaccination rates. The lived experiences and personal connections of SACTF members in the Peel region along with their adoption of a people-centred care approach facilitated direct engagement and collaboration with the community that they were serving. This ensured that their efforts at public dissemination of COVID-19 information were culturally apt, linguistically varied, and appealing to diverse demographics. They disseminated information using modalities familiar to the community, such as ethnic media and WhatsApp.

The interviews and focus group reflect SACTF’s frustration with the negative portrayal of racialized communities in mainstream media that often blamed them for the spread of infection and higher mortality [27,36]. SACTF advocacy shifted the media’s gaze from South Asian cultural and religious gatherings as contributing to COVID-19 spread in Brampton (and elsewhere) to the structural inequities that increased the vulnerability of South Asians to COVID-19 infections. South Asian-predominated regions–such as Brampton–already had per capita lower health resources allocated to them than other pre-pandemic areas of the country [12]. Brampton Mayor Patrick Brown has noted this in numerous media briefings [33]. This contributed directly to resource shortages (such as for Personal Protective Equipment, PPE) and reduced medical professional availability, leading to long wait times for regular healthcare needs and longer wait times for non-specialized services. SACTF’s advocacy drew attention to the need for equitable investment in healthcare delivery to build a resilient health system that can serve the needs of Canadians beyond immediate crisis management.

The lack of flexibility/adaptability of the existing healthcare systems, inadequate healthcare delivery to areas dominated by racialized population, lack of access to public health information in South Asian languages with cultural relevance, and lack of dissemination of information using tools that are already familiar within the community acted as barriers to following healthcare guidelines. SACTF knowledge dissemination materials illustrate the critical role of communicating public health messages in user-friendly language and using culturally appropriate tools in increasing compliance during a health crisis, such as a pandemic. This is especially pertinent to newcomers to Canada, international students, and older adults who may not have adequate fluency in English or French. COVID-19 health guidelines were not advertised familiarly [5] and the use of medical jargon and uncommon terms made the information even more difficult to grasp for those lacking fluency in English or French. Public health messaging also changed quickly throughout the COVID-19 pandemic as new research data, treatment procedures, and vaccines became available, making it more difficult for the public to stay updated with the latest guidelines. Mere translation of health messaging into other South Asian languages is also not sufficient when the Western Canadian experience is at the core of these messages, rendering the materials inaccessible to other linguistic communities [5]. SACTF videos and skits in South Asian languages offered the community guidance on how to comply with healthcare guidelines within their ethno-cultural contexts by suggesting possible ways of navigating everyday scenarios such as politely declining invitations for tea during lockdowns. SACTF health promotional materials, many of which went viral, illustrate how supporting community groups to develop their own health promotion campaigns can effectively enhance public engagement of government health agencies.

The gaps and challenges in understanding the South Asian community’s specific health, cultural, and socio-economic vulnerabilities and needs during the pandemic was evident from the initial public health response. SACTF’s role in bridging that gap by educating South Asian communities on public health guidelines shows that public health agencies can benefit from partnering with community groups to (re)build trust with marginalized populations. During the COVID-19 pandemic, the underlying distrust of racialized communities to Western health messaging [7,37], was exacerbated by a slew of misinformation on digital media. In addition, with COVID-19-related healthcare services and guidelines changing rapidly as the pandemic evolved, communities benefited from advocacy groups and racialized healthcare professionals stepping in to support their communities.

Healthcare studies have shown that diversity in the healthcare workforce can improve patient experiences and clinical outcomes [38]. Further, evidence suggests that when patients see their race represented in their healthcare providers, they are more likely to have positive care experiences [39]. It is SACTF’s close ties with the community that allowed them to identify effective ways to engage with diverse members of the South Asian community, including seniors, newcomers, international students, and those with co-morbidities, and mitigate the adverse health impacts of the pandemic on this marginalized group. By advocating for the centering of the voices and varied experiences of marginalized populations in health systems decision-making processes, advocacy groups such as SACTF emerged as critical to both pandemic response and recovery.

Our study, therefore, points to the need for adequate representation of racialized healthcare professionals and researchers in decision-making processes. SACTF’s public engagement approach demonstrates that the knowledge and lived experiences of healthcare workers can be a resource for the government and its agencies to improve healthcare delivery and health outcomes for racial minorities. For example, SACTF was alert to the multiple axes of inequalities within the South Asian community and they addressed the differential impacts arising from the interplay of multiple factors such as race, age, status in Canada, and employment, among others. This attention to intersectionality [40,41] is evident, from their alertness to age-related media use. They created public health messages in various South Asian languages to reach out to older adults lacking fluency in the official languages and employed different social media platforms to reach different South Asian demographics (for example, WhatsApp for older adults, TikTok for youth). Rather than providing community members with the information produced by the health system, they crafted the information in ways that appealed to the sensibilities of the South Asian community, which in turn ensured better reception of healthcare information within a community that government agencies were finding difficult to reach. This suggests that healthcare systems can benefit diverse communities when they consider racialized healthcare workers as active stakeholders who have the ability to produce and distribute health information in ways accessible to diverse communities.

SACTF’s timely intervention included making public health guidelines accessible to South Asian populations through culturally appropriate, multimodal health information sharing, alleviating distrust in healthcare, fighting misinformation, improving testing accessibility, and increasing vaccination in their community. This illuminates that healthcare professionals from racially marginalized groups who share the lived experiences of the population they serve can offer critical insights on the needs of their communities and the structural inequities that hinder the wellbeing of their communities. It is evident from SACTF advocacy and its impact that racialized healthcare workers can provide culturally sensitive care by recognizing and filling gaps in healthcare policy and thereby improve the public’s adherence to healthcare guidelines (e.g. masking, isolation, and vaccination, in the case of COVID-19). Their success in public outreach proves what many researchers and community activists have been saying: "Along with the science, it is imperative to understand cultures, values, languages, histories, and other determinants of human behaviour" [25]. That is, public health interventions have to be tailored to the specific needs and contexts of diverse communities. As the COVID-19 pandemic makes it clear biomedical solutions cannot resolve misinformation or vaccine hesitancy. However, policymakers can harness the cultural insider knowledge and lived experiences of its racialized healthcare workers in planning and designing public health activities to achieve better health outcomes.

## Conclusion

This study argues for the incorporation of the perspectives, knowledge, and experiences of racialized healthcare workers into decision-making processes to build a more equitable and effective healthcare system. Understanding the potential impact of the public engagement approaches of advocacy groups such as SACTF from the perspectives of the SACTF Board of Directors is a first step. Further research is warranted, particularly focusing on engaging key stakeholders in the process. Carefully considering critiques and assessments of public health policies by prioritizing the voices of health professionals with firsthand experiences in racialized communities and patient communities is essential if we are to address structural global health inequities and improve health outcomes for marginalized communities. A one-size-fits-all approach falls short in the face of diverse communities, and health systems need to display both flexibility and cultural sensitivity in their public health strategies. Collaboration with community advocacy groups can offer public health agencies and policymakers on-the-ground assessments by racialized healthcare workers, which can help to improve healthcare delivery. Providing resources to such advocacy groups will allow them to pivot quickly to address emerging health issues and work in partnership with public health institutions, government agencies, non-profit organizations, and researchers. They can effectively serve as allies and trusted sources for both the community and the government both in times of calm and in times of crisis.

## Supporting information

S1 Data Thematic analysis. (XLSX)

## References

[1] Jung A-S, Haldane V, Neill R, Wu S, Jamieson M, Verma M, et al. National responses to covid-19: drivers, complexities, and uncertainties in the first year of the pandemic. BMJ. 2021; e068954. doi: 10.1136/bmj-2021-068954 34840138 PMC8624066

[2] Bhatia R. Public engagement is key for containing COVID-19 pandemic. Indian J Med Res. 2020;151: 118–120. doi: 10.4103/ijmr.IJMR_780_20 32242877 PMC7357402

[3] Yuan M, Lin H, Wu H, Yu M, Tu J, Lü Y. Community engagement in public health: a bibliometric mapping of global research. Arch Public Health. 2021;79: 6. doi: 10.1186/s13690-021-00525-3 33436063 PMC7801880

[4] Nana-Sinkam P, Kraschnewski J, Sacco R, Chavez J, Fouad M, Gal T, et al. Health disparities and equity in the era of COVID-19. J Clin Transl Sci. 5: e99. doi: 10.1017/cts.2021.23 34192054 PMC8167251

[5] Kandasamy S, Ariyarajah A, Limbachia J, An D, Lopez L, Manoharan B, et al. South Asian youth as vaccine agents of change (SAY-VAC): evaluation of a public health programme to mobilise and empower South Asian youth to foster COVID-19 vaccine-related evidence-based dialogue in the Greater Toronto and Hamilton Area, Canada. BMJ Open. 2022;12: e061619. doi: 10.1136/bmjopen-2022-061619 36153036 PMC9511009

[6] Peel Region, South Asian community disproportionately hit during first year of COVID-19: study | Globalnews.ca. [cited 24 Jun 2024]. Available: https://globalnews.ca/news/8967841/peel-region-south-asian-community-covid-19-impact-study/.

[7] Ben J, Cormack D, Harris R, Paradies Y. Racism and health service utilisation: a systematic review and meta-analysis. PLOS ONE. 2017;12: e0189900. doi: 10.1371/journal.pone.0189900 29253855 PMC5734775

[8] Immigration & Ethnocultural Diversity. In: GeoHub [Internet]. 2019. Available: https://geohub.brampton.ca/pages/profile-diversity.

[9] 2021 Census data on ethnic diversity, religion, and immigration—Region of Peel. [cited 16 Feb 2024]. Available: https://www.peelregion.ca/articles/2022/census-data-ethnic-diversity-religion-immigration.asp.

[10] Guttmann A, Gandhi S, Wanigaratne S, Lu H, Ferreira-Legere LE, Paul J, et al. COVID-19 in immigrants, refugees and other newcomers in Ontario: characteristics of those tested and those confirmed positive, as of June 13, 2020. ICES; 2020. Available: https://www.ices.on.ca/publications/research-reports/covid-19-in-immigrants-refugees-and-other-newcomers-in-ontario-characteristics-of-those-tested-and-those-confirmed-positive-as-of-june-13-2020/.

[11] Grant K. Data show poverty, overcrowded housing connected to COVID-19 rates among racial minorities in Toronto. The Globe and Mail. 2 Jul 2020. Available: https://www.theglobeandmail.com/canada/toronto/article-data-show-poverty-overcrowded-housing-connected-to-covid-19-rates/. Accessed 4 Jan 2024.

[12] Gupta TD, Nagpal S. Unravelling discourses on COVID-19, South Asians and Punjabi Canadians. Stud Soc Justice. 2022;16. Available: https://journals.library.brocku.ca/index.php/SSJ/article/view/3471.

[13] Council of Agencies Serving South Asians (CASSA), South Asian Legal Clinic of Ontario (SALCO), South Asian Women’s Rights Organization (SAWRO). The impact of COVID-19 on South Asians in Canada–SALCO. 1 May 2020 [cited 7 Nov 2023]. Available: https://salc.on.ca/the-impact-of-covid-19-on-south-asians-in-canada/.

[14] COP-COC. Reconstruction and Reset Plan for Canada. In: Colour of Poverty—Colour of Change [Internet]. 8 Sep 2020 [cited 7 Nov 2023]. Available: https://colourofpoverty.ca/2020/09/08/cop-coc-reconstruction-and-reset-plan-for-canada/.

[15] Kandasamy S, Manoharan B, Khan Z, Stennett R, Desai D, Nocos R, et al. Perceptions of COVID-19 risk, vaccine access and confidence: a qualitative description of South Asians in Canada. BMJ Open. 2023;13: undefined-undefined. doi: 10.1136/bmjopen-2022-070433 37015794 PMC10083522

[16] Bhalla M, Boutros H, Meyer SB. Aunties, WhatsApp, and “haldi da doodh”: South Asian communities’ perspectives on improving COVID-19 public health communication in Ontario, Canada. Can J Public Health Rev Can Sante Publique. 2022;113: 46–53. doi: 10.17269/s41997-022-00712-x 36449223 PMC9713156

[17] Pringle W, Sachal SS, Dhutt GS, Kestler M, Dubé È, Bettinger JA. Public health community engagement with Asian populations in British Columbia during COVID-19: towards a culture-centered approach. Can J Public Health Rev Can Sante Publique. 2022;113: 14–23. doi: 10.17269/s41997-022-00699-5 36329357 PMC9633035

[18] COVID-19 vaccination coverage by ethnicity: Insight from the Canadian Community Health Survey (CCHS). In: Government of Canada [Internet]. 17 Jun 2022 [cited 2 Nov 2023]. Available: https://www.canada.ca/en/public-health/services/immunization-vaccines/vaccination-coverage/covid-19-vaccination-coverage-ethnicity-insight-canadian-community-health-survey.html.

[19] Dhamanaskar R, Abelson J. Engaging the public in pandemic policy response: missed and future opportunities for Canada. In: Public Engagement in Health Policy [Internet]. 15 Aug 2023 [cited 11 Mar 2024]. Available: https://www.engagementinhealthpolicy.ca/blog/engaging-the-public-in-pandemic-policy.

[20] South Asian Community Health Task Force. In: SA Comm. Task Force [Internet]. 2023 [cited 2 Nov 2023]. Available: https://www.southasianctf.ca/about-us.

[21] Nasser S. Brampton has emerged as one of Ontario’s COVID-19 hotspots, but experts urge caution on where to lay blame. CBC News. 14 Sep 2020. Available: https://www.cbc.ca/news/canada/toronto/brampton-coronavirus-covid-19-south-asian-1.5723330.

[22] Demographics. [cited 16 Feb 2024]. Available: https://data.peelregion.ca/pages/demographics.

[23] Census Information Hub. [cited 16 Feb 2024]. Available: https://census-regionofpeel.hub.arcgis.com/.

[24] Hinchliffe S, Jackson MA, Wyatt K, Barlow AE, Barreto M, Clare L, et al. Healthy publics: enabling cultures and environments for health. Palgrave Commun. 2018;4: 57. doi: 10.1057/s41599-018-0113-9 29862036 PMC5978671

[25] Jones E, Wright JM, Holmes B, Goldenberg MJ, Dyck E, Bagshaw SM. The humanities and health policy. 2023 Nov. Available: https://rsc-src.ca/en/research-and-reports/covid-19-policy-briefing/humanities-and-health-policy.

[26] Anderson KJ. Vancouver’s Chinatown: racial discourse in Canada, 1875–1980. McGill-Queen’s University Press; 1991. Available: https://www.jstor.org/stable/j.ctt80533.

[27] Wallace SI. Not fit to stay: public health panics and South Asian exclusion. UBC press; 2017.

[28] Mawani R. Colonial proximities: crossracial encounters and juridical truths in British Columbia, 1871–1921. In: UBC Press [Internet]. UBC Press; 2009 [cited 4 Jan 2024]. Available: https://www.ubcpress.ca/colonial-proximities.

[29] Shah N. Contagious divides: epidemics and race in San Francisco’s Chinatown. 1st ed. University of California Press; 2021. doi: 10.2307/j.ctv1gwqmp3 12395801

[30] Abelson J, Montesanti S, Li K, Gauvin F-P, Martin E. Effective strategies for interactive public engagement in the development of health care policies and programs. Can Health Serv Res Found. 2010.

[31] Yin RK. Designing case studies: identifying your case(s) and establishing the logic of your study. 4th ed. Case Study Research: Design and Methods. 4th ed. Los Angeles: SAGE Publications,Inc; 2009.

[32] Thompson N. Coronavirus: spread of COVID-19 in Brampton linked to systemic factors, experts say—Toronto | Globalnews.ca. In: Globalnews [Internet]. 29 Nov 2020 [cited 27 Dec 2023]. Available: https://globalnews.ca/news/7491119/brampton-coronavirus-covid-19-systemic-factors/.

[33] Fox C. Peel hospitals “starting to provide care in non-traditional spaces” amid influx of COVID-19 patients: Loh. In: CP24 [Internet]. 2 Dec 2020 [cited 4 Jan 2024]. Available: https://www.cp24.com/news/peel-hospitals-starting-to-provide-care-in-non-traditional-spaces-amid-influx-of-covid-19-patients-loh-1.5213731.

[34] Elghawaby A. Pandemic public health messaging isn’t on WhatsApp and that’s hurting some communities. In: Toronto Star [Internet]. 15 Dec 2020 [cited 27 Nov 2023]. Available: https://www.thestar.com/opinion/contributors/pandemic-public-health-messaging-isn-t-on-whatsapp-and-that-s-hurting-some-communities/article_88c5714c-bb0e-5c99-92e2-51416ed97edc.html.

[35] Racialized minorities. [cited 1 Jul 2024]. Available: https://www.thecanadianencyclopedia.ca/en/article/racialized-minorities.

[36] Paradkar S. Covidiots come in all colours. Using race-based data to demonize South Asians is a cruel twisting of the evidence. In: Toronto Star [Internet]. 18 Nov 2020 [cited 8 Jan 2024]. Available: https://www.thestar.com/opinion/star-columnists/covidiots-come-in-all-colours-using-race-based-data-to-demonize-south-asians-is-a/article_bf8c0af6-c2a7-57c6-925b-5f37ac2bf1c0.html.

[37] Mahabir DF, O’Campo P, Lofters A, Shankardass K, Salmon C, Muntaner C. Experiences of everyday racism in Toronto’s health care system: a concept mapping study. Int J Equity Health. 2021;20: 74. doi: 10.1186/s12939-021-01410-9 33691682 PMC7943708

[38] Gomez LE, Bernet P. Diversity improves performance and outcomes. J Natl Med Assoc. 2019;111: 383–392. doi: 10.1016/j.jnma.2019.01.006 30765101

[39] Shen MJ, Peterson EB, Costas-Muñiz R, Hernandez MH, Jewell ST, Matsoukas K, et al. The effects of race and racial concordance on patient-physician communication: a systematic review of the literature. J Racial Ethn Health Disparities. 2018;5: 117–140. doi: 10.1007/s40615-017-0350-4 28275996 PMC5591056

[40] Crenshaw K. Mapping the margins: Intersectionality, identity politics, and violence against women of color. Stanford Law Rev. 1991;43: 1241–1299. doi: 10.2307/1229039

[41] Maestripieri L. The Covid-19 pandemics: why intersectionality matters. Front Sociol. 2021;6: 642662. doi: 10.3389/fsoc.2021.642662 33869589 PMC8022485

